# Proximate determinants of infant mortality in Ethiopia, 2016 Ethiopian demographic and health surveys: results from a survival analysis

**DOI:** 10.1186/s13690-019-0387-4

**Published:** 2020-01-22

**Authors:** Masrie Getnet Abate, Dessie Abebaw Angaw, Tamrat Shaweno

**Affiliations:** 1 Biostatistics Unit, Epidemiology Department, Institute of Health, Jimma University, Jimma, Ethiopia; 2 Biostatistics and Epidemiology Department, Institute of Public Health college of medicine and health science, University of Gondar, Gondar, Ethiopia; 3 Epidemiology Department, Institute of Health, Jimma University, Jimma, Ethiopia

**Keywords:** Infant mortality, Cox regression hazard model, Ethiopia

## Abstract

**Background:**

In Ethiopia, large scale health care efforts had been done to promote infant health and survival. However, nationwide data is lacking on the survival status and proximate determinants of infant mortality in Ethiopia. Therefore, this study was aimed to identify the survival status and determinants of infant mortality in Ethiopia using Ethiopian Demographic and Health Survey (EDHS).

**Methods:**

The data source for this study was the 2016 Ethiopian Demographic and Health Survey. Records of all 10,641 live births and survival informations of all 2826 infants born 5 years before the survey were reviewed. Kaplan-Meier method and Cox proportional hazards regression model were employed to identify the proximate determinants associated with the infant mortality.

**Results:**

The results of Kaplan-Meier estimation showed that the highest infant deaths occurred in the early months of life immediately after birth and declined in the later months of follow-up time. About 65% of infant deaths occurred during the first month’s of life. Using the Cox proportional hazard model we found that: mothers’ level of education, preceding birth interval, plurality, size of child at birth and sex of child as significant predictors of infant mortality. The risk of dying in infancy was lower for babies of mothers with secondary education (RR = 0.68, 95% CI: 0.56–0.98), higher education (RR = 0.51, 95% CI:0.45–0.80), for preceding birth interval longer than 47 months (RR = 0.51, 95% CI: 0.27, 0.92) and higher for birth interval shorter than 24 months (RR = 2.02, 95% CI:1.40–2.92), for multiple births (RR = 4.07, 95% CI: 1.14–14.50), for very small size of infants (RR = 3.74, 95% CI:1.73–8.12), for smaller than average size infants (RR = 3.23, 95% CI: 1.40–7.41) and for female infants (RR = 1.26, 95% CI: 1.01–1.56) compared to the reference category.

**Conclusions:**

A significant proportion of infants died during the study period of which nearly two third of deaths occurred during the first months of life. Thus, close monitoring and supporting reproductive age mothers to increase the uptakes of family planning and antenatal care and follow-up is highly recommended to increase the infant survival.

## Background

The infant mortality rate is a key population health indicator [1]. A high infant mortality rate can reflect poor quality of care and/or lack of access to care [2]. The sustainable development goal (SDG) target for child mortality aims to end, by 2030, preventable deaths of new borns and children under 5 years of age with all countries aiming to reduce neonatal mortality to at least as low as 12 deaths per 1000 live births and under five mortality to at least as low as 25 deaths per 1000 live births [3].

In 2016, infant mortality contributed to more than 75% of all underfive deaths globally [4]. For the last two decades, infant and child survival has remained an upmost global priority. To reduce infant deaths, massive investment has been done including an access to improved basic health care, under-five nutrition, personal hygiene and environmental sanitation, and uptakes of breast feeding and vaccination [4]. Accordingly, all regions of the world have shown declines in IMR and under-five mortality rate has shown declines in IMR and under-five mortality rate over the last two decades [4, 5]. Conversely, these achievements are confronted by inequalities that persist among regions and within countries [4]. The economically disadvantaged countries and populations continue to bear the substantial burden of infant deaths [4].

The risk of a child dying before his first birthday is higher in African region (52 per 1000 live births), over six times higher than that in the European region (8 per 1000 live births) [6]. In Africa, the main risk factors associated with a high number of infants deaths include: lack of access to funds and infrastructure, access to education, lack of medical professionals, poverty, and discrimination [4, 6]. In addition, there is a higher prevalence in the region of those diseases that are infants are particularly vulnerable to such as: pneumonia, diarrhea, malnutrition, asphyxia, birth complications, and malaria [6]. In addition, infant mortality rates vary across income groups, by level of education, based on residence, maternal child bearing age, gender, ANC (Antenatal care) follow-up status, and geographical areas [6, 7].

Ethiopia is among the few regions with the highest burden of IMR. To reverse this, Ethiopia has made large-scale investments in basic healthcare over the last decade; mainly in the disadvantaged rural population [7–9]. Antenatal care (ANC) attendance, access to primary health care, coverage of fully vaccinated children and skilled deliveries have shown dramatic improvements including maternal health services being delivered free of charge at every health facilities [9]. In response to these various interventions, Ethiopia has shown a significant decline in infant and child deaths; and is among the few countries which achieved millennium development goal-4 in 2013 [10]. Recent estimates reported that IMR has dropped from 121 per 1000 live births in 1990 to below 40 per 1000 live births in 2017 [10].

The 2016 Ethiopian Demographic and Health Survey (EDHS) result showed that the infant mortality rate for the 5 years before the survey is 48 deaths per 1000 live births. The survey’s results showed that infant mortality declined from 97 deaths per 1000 live births in 2000 to 48 deaths per 1000 live births in 2016, which accounts for a 50% reduction in the last 16 years. Among those infants that do not survive, about 72% deaths occur before the first birthday [8]. However, population based data is limited on the current infant survival status and predictors of mortality among infants in Ethiopia. A better understanding of the time when and why infant death occurs and proximate determinants of infant mortality in Ethiopia is crucial to design time relevant intervention strategies that will contribute to reducing the burden of infant mortality in Ethiopia. Thus, the aim of this study was to assess the proximate, distal and intermediate determinants of infant mortality in Ethiopia.

## Methods

### Study design and data set

The data set used in this study was obtained from the Ethiopian Demographic and Health Survey conducted in 2016, which is the fourth comprehensive survey. It was a population-based cross-sectional study conducted from January 18, 2016 to June 27, 2016, across the country and it is available from the MEASURE DHS database at https://dhsprogram.com/data/available-datasets.cfm. The 2016 EDHS sample was selected in two stages. In the first stage, the total of 645 clusters (202 in urban and 443 in rural) were randomly selected proportional to the household size from the sampling strata and in the second stage, 28 households per cluster were selected using systematic random sampling.

Representative samples of 18,008 households were selected and 16, 650 households were interviewed in 2016 EDHS. For individual interview, 16, 583 eligible women were identified from the interviewed household. A total of 15,683 women aged 15–49 interviews were completed [8]. A total of 10, 641 live births during the 5 years preceding the survey were considered to calculate the IMR. Accordingly, 2628 infants born between 2011 and 2015 and followed up for 1 year during the 5 years preceding the survey were included into the study. The denominator in all analyses as 2628, which corresponds to the total number of infants born between 2011 and 2015 and followed up for 1 year, except for three variables including preceding birth interval, size of child at birth and infant breastfed status at birth. Accordingly, the denominator for the variable “birth interval”, was 2057, because a total of 571 study participants having only one child (the current infant) were excluded from the analysis for birth interval. Similarly, 41 for the variable “size of child at birth” and 130 participants for the variable “infant breastfed status at birth” respectively were excluded from the analysis mainly due to their unknown status about size of the child at birth and infant breast fed status at birth.

### Study variables

In this study, the major outcome variable was time to infant death, measured in months between birth and the first birthday within the preceding 5 years of the survey. In this finding, those infants who died between birth and the first birthday were events and those who were still alive and did not reach their first birthday were considered as censored. Death was considered as the death of infant during the 5 years preceding the survey due to any cause.

### Predictor variables

**Fig. 1:**
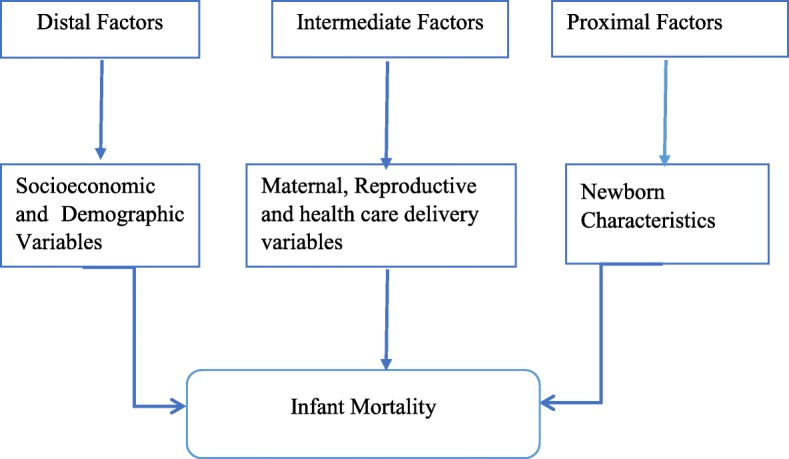
Conceptual framework of infant mortality in Ethiopia adapted form Mosley and Chen (1984)

The predictor variables were categorized into three (distal factors, intermediate factors and proximal factors) based on Mosley and Chen (1984) theoretical framework showed as in Fig. 1 [11]. Accordingly, proximal vs intermediate vs distal factors were defined as background variables that put influence on infant mortality directly, intermediately and remotely, respectively. For distal factors we considered: educational level of mother’s (no education, primary, secondary, higher), household wealth (poorest, poorer, middle, richer, richest), sex of household head (male, female), region (Tigray, Afar, Amhara, Oromia, Somalia, Benishangul, SNNPR, Gambela, Harari, Diredawa, Addis Ababa) and place of residence (urban, rural). Similarly, variables considered as intermediate factors included: age of mother’s at first birth (< 20,20–30,> 30), birth order (< 3, 3–4,> 4), place of delivery (home, health institutions), preceding birth interval in months (< 24, 24–47,> 47), number of antenatal care visits (< 4, ≥4) and smoking cigarettes during pregnancy (yes, no). Variables considered as proximal factors included: plurality (single, multiple), size of child at birth (large, larger than average, average, smaller than average, very small), sex of child (male, female), and breastfed upon birth (immediately, not immediately) were considered as predictor variables of infant mortality. Baby’s size at birth (birth weight) was assessed by monther’s self-report. To reduce the recall bias; mother’s age (young mothers have good recall) /level of education (more educated mother’s have good recall) and told from physicians during birth were considered. Mothers who “didn’t know or could not estimate their infant’s birth weight were excluded. Accordingly, baby’s birth size was categorized as (1 = very large (> 3500 g); (2 = larger than average (3500–3000 g); (3 = average (2500 to 3000 g); (4 = small (2500-2000 g); and (5 = very smaller than average (< 2500 g)). Similarly, with regard to wealth index, households were given scores based on the number and kinds of consumer goods they own, ranging from a television to a bicycle or car, in addition to housing characteristics such as source of drinking water, toilet facilities, and flooring materials. These scores are derived using principal component analysis [8]. National wealth quintiles were compiled by assigning the household score to each usual (de jure) household member, ranking each person in the household population by her or his score, and then dividing the distribution into five equal categories, each comprising 20% of the population. Accordingly, the household wealth index was categorized as (1 = Poorest; 2 = Poorer; 3 = Middle; 4 = Richer; 5 = Richest).

### Statistical analysis

We used Cox regression model in the survival analysis, statistical analyses were performed using STATA version 14. Cox regression model is the most popular regression techniques for survival analysis because it examines the impact of various predictors of the risk of death. The additional advantage of this model is that it accounts for censoring in the data [12, 13]. Variables with a *p*-value < 0.25 in the univariate Cox regression analysis were included in the multivariate analysis. We estimated hazard ratios and their 95% confidence intervals. In the multivariate analysis, we used *p* < 0.05 as threshold for statistical significance.

Infant mortality rates were calculated by summing up the number of infant deaths during infancy period divided by the total number of months of follow-up during the study period. Performing log-log survival curves based on Schoenfeld residuals was used to assess the proportional hazard assumption. Accordingly, the model fitness statistics was significant (*P* = 0.0137). Variables with missing/don’t know category were excluded from analysis. Accordingly, the rate of missing data for the three variables was below 2%.

The Kaplan-Meier survival table was used to compare the survival experiences of infants separated by significant predictor variables at child birth. Incidence density was calculated for events (deaths) using infant months of observation.

## Results

### Descriptive results

A total of 10, 641 live births born during the 5 years preceding the survey and who had a full 1 year follow-up time were included into this analysis. The male to female sex composition at birth was nearly the same and the great majorities (96%) were single births (Table 1). From a total of 2628 infants included into this study, approximately four-fifth (81%) were born to a rural community and almost 31% of infants were born with size at birth below the average infant size at birth (Table 1).

**Table 1:** Summary results of covariates of time-to-death for infants in Ethiopia, 2016 Ethiopian Demographic and Health Survey

Characteristics/Variables	Categories	Status of infants	
		Event (deaths) (%)	Censored (alive) (%)
A. Distal/Socioeconomic factors			
Maternal level of education (*N* = 2628)	No education	347 (13.2)	1229 (46.8)
	Primary	117 (4.5)	617 (23.5)
	Secondary	32 (1.2)	190 (7.2)
	Higher	6 (0.2)	90 (3.4)
Household wealth (In quintiles) (*N* = 2628)	Poorest	219 (8.3)	757 (28.8)
	Poorer	92 (3.5)	347 (13.2)
	Middle	62 (2.4)	292 (11.1)
	Richer	64 (2.4)	261 (9.9)
	Richest	65 (2.5)	469 (17.8)
Region (*N* = 2628)	Tigray	36 (1.4)	221 (8.4)
	Afar	55 (2.1)	205 (7.8)
	Amhara	39 (1.5)	198 (7.5)
	Oromia	68 (2.6)	333 (12.7)
	Somali	95 (3.6)	303 (11.5)
	Benishangul	47 (1.8)	164 (6.2)
	SNNPR*	56 (2.1)	250 (9.5)
	Gambela	33 (1.3)	126 (4.8)
	Harari	32 (1.2)	124 (4.7)
	Drie Dawa	27 (1.0)	98 (3.7)
	Addis Ababa	14 (0.5)	104 (4.0)
Place of residence (*N* = 2628)	Urban	60 (2.3)	430 (16.4)
	Rural	442 (16.8)	1696 (64.5)
Sex of household head (*N* = 2628)	Male	404 (15.4)	1682 (64.0)
	Female	98 (3.7)	444 (16.9)
B. Intermediate/Mother’s characteristics			
Preceding birth interval (in months) (*N* = 2057)	< 24	160 (7.8)	312 (15.2)
	24–47	159 (7.7)	886 (43.1)
	> 47	67 (3.3)	473 (23.0)
Maternal age at first birth (in years) (N = 2628)	< 20	329 (12.5)	1254 (47.7)
	20–30	165 (6.3)	851 (32.4)
	> 30	8 (0.3)	21 (0.8)
Birth order (N = 2628)	< 3	191 (7.3)	836 (31.8)
	3–4	121 (4.6)	564 (21.5)
	> 4	190 (7.2)	726 (27.6)
Place of delivery (N = 2628)	Home	372 (14.2)	1233 (46.9)
	Health Institution	130 (4.9)	893 (34.0)
Number of antenatal care visits (N = 2628)	< 4	466 (17.7)	1984 (75.5)
	≥ 4	36 (1.4)	142 (5.4)
Smoking cigarettes during pregnancy (N = 2628)	Yes	493 (18.8)	2119 (80.6)
	No	9 (0.3)	7 (0.3)
C. Proximal/child’s characteristics			
Plurality (N = 2628)	Single birth	449 (17.1)	2079 (79.1)
	Multiple births	53 (2.0)	47 (1.8)
Size of child at birth (*N* = 2587)	Very large	96 (3.7)	286 (11.1)
	Larger than average	53 (2.0)	263 (10.2)
	Average	170 (6.6)	917 (35.4)
	Smaller than average	43 (1.7)	203 (7.8)
	Very small	124 (4.8)	432 (16.7)
Sex of child (N = 2628)	Male	309 (11.8)	1052 (40.0)
	Female	193 (7.3)	1074 (40.9)
Infant breastfed status at birth (*N* = 2498)	Immediately	264 (10.6)	1438 (57.6)
	Not immediately	139 (5.6)	657 (26.3)

*SNNPR Southern Nations Nationalities and Peoples region, *EDHS* Ethiopian Demographic and Health Survey

### Infant survival status

**Fig. 2:**
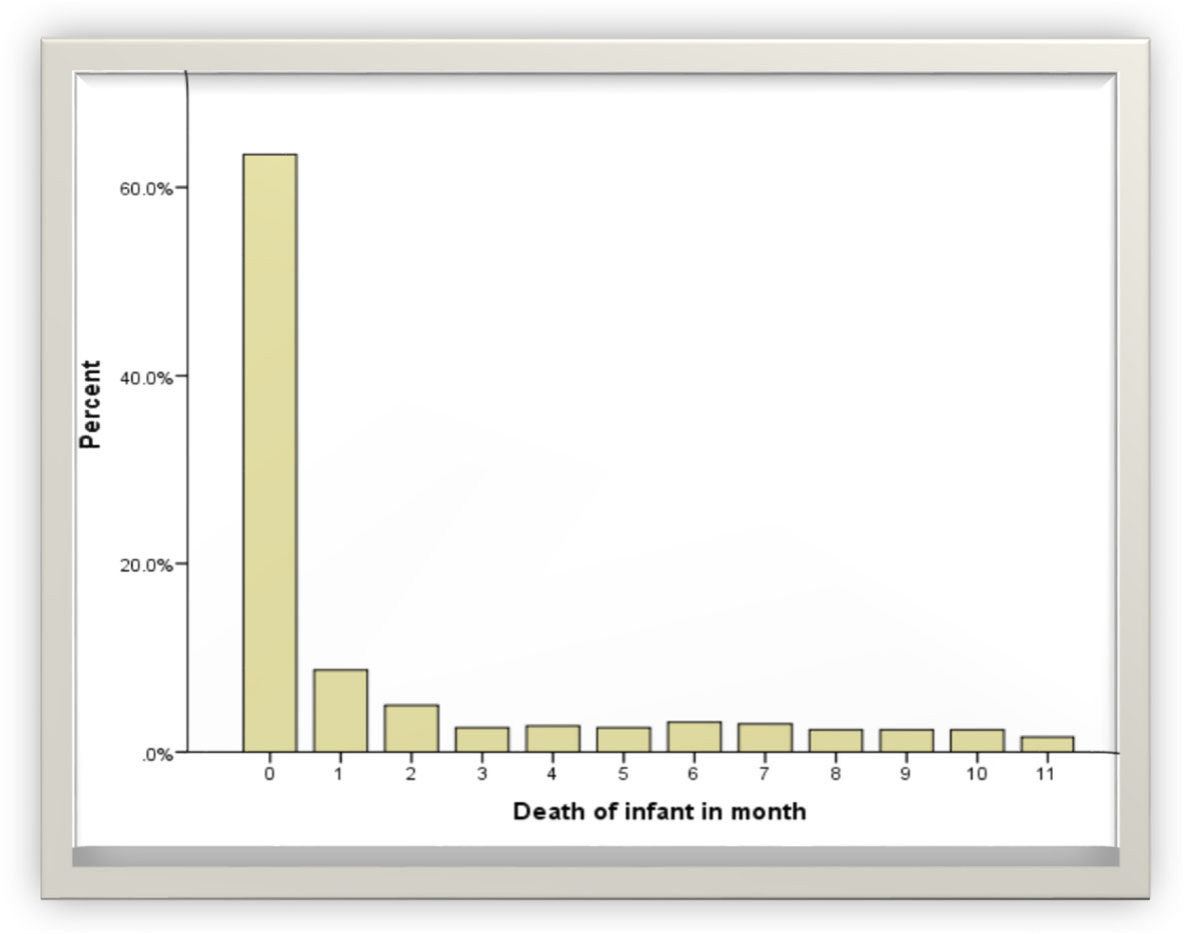
The overall Kaplan-Meier estimates of infant mortality in Ethiopia measured in months, using 2016 Ethiopian Demographic and Health Survey

From a total of 2628 infants followed for a full 1 year time period, the cumulative survival probability at the end of first year was 95.2% with the median survival time of 6 months ((SD = 0.11), (95%CI, 7.78–6.22)). The difference in survival probability between male and female infants was statistically significant. Among the infants who failed to survive up to their first years of age (4.8%), nearly two-third (65%) had died during the first months of life (Fig. 2). The infant survival probability varied by maternal level of education, preceding birth intervals, plurality, size of infant at birth and sex of infant.

### Predictors of infant mortality

Maternal level of educational, household wealth index, place of residence, preceding birth interval, age of mother at first birth, birth order, place of delivery, number of antenatal care visits, smoking status of cigarettes during pregnancy, plurality, size of infants at birth, breastfed status at birth and sex of the child were included in the multivariate cox regression. In the multivariate cox model, maternal level of education, preceding birth intervals, plurality, size of infant at birth and sex of the child showed significant association in the final multivariate cox regression model (Table 2).

**Table 2:** Relative risk (RR) of infant mortality. Results of a multivariate Cox’s Proportional Hazard regression, 2016 Ethiopian Demographic and Health Survey

Variables	Categories	Estimated RR	95% CI for RR
A. Distal/socioeconomic factors			
Maternal level of education	No education ^(ref)^	1	
	Primary	0.95	0.59–2.62
	Secondary	0.68	0.56–0.98
	Higher	0.51	0.45–0.80
Household wealth (in quintiles)	Poorest	1.68	0.69–4.11
	Poorer	1.36	0.51–3.62
	Middle ^(ref)^	1	
	Richer	1.64	0.59–4.55
	Richest	2.45	0.78,-7.71
Place of residence	Urban ^(ref)^	1	
	Rural	1.80	0.501–6.40
B. Intermediate/mother’s characteristics			
Preceding birth interval (in months)	< 24	2.02	1.40–2.92
	24–47 ^(ref)^	1	
	> 47	0.51	0.27–0.92
Maternal age at first birth (in years)	< 20	1.23	0.84–1.85
	20–29 ^(ref)^	1	
	> 30	0.70	0.60–1.93
Birth order	< 3	0.85	0.38,-1.90
	3–4 ^(ref)^	1	
	> 4	0.75	0.39–1.42
Place of delivery	Home	1.90	0.78–4.62
	Health Institution ^(ref)^	1	
Number of antenatal care visits	< 4 ^(ref)^	1	
	≥ 4	0.63	0.56–1.45
Smoking cigarettes during pregnancy	No ^(ref)^	1	
	Yes	1.28	0.60–2.78
C. Proximal/child’s characteristics			
Plurality	Single birth ^(ref)^	1	
	Multiple birth	4.07	1.14–14.5
Birth weight categories (in gm)	Very large	2.02	0.56–7.34
	Larger than average	1.76	0.60–5.15
	Average ^(ref)^	1	
	Smaller than average	3.23	1.40–7.41
	Very small	3.74	1.73–8.12
Breastfed at birth	Immediately ^(ref)^	1	
	Not immediately	1.13	0.63–2.04
Sex of child	Male	1.26	1.01–1.56
	Female ^(ref)^	1	

*(ref)* the reference category, *gm* gram

### Distal factors/socioeconomic characteristics

In comparison to mothers who had no education (the reference category), the estimated hazard ratios for mothers who had secondary and higher education were 0.68 (95% CI: 0.56–0.98) and 0.51 (95% CI: 0.45, 0.80) respectively. The risk of death was lower by 32 and 49% for infants born from mothers who had secondary and higher education respectively, compared with infants born from non-educated mothers (Table 2).

### Intermediate factors/mother’s characteristics

The estimated hazard ratio for infants both with a preceding birth interval shorter than 24 months was 2.11 (95% CI: 1.67–2.66) and for longer than 47 months was 0.51 (95% CI: 0.27–0.92) compared to birth interval between 24 and 47 months (reference category). The risk of death of infants with a preceding birth interval shorter than 24 months was 2.11 times higher and for longer than 47 months reduced the risk of death of infants by 49% compared to the reference category (Table 2).

#### Proximal factors/child’s characteristics

**Fig. 3:**
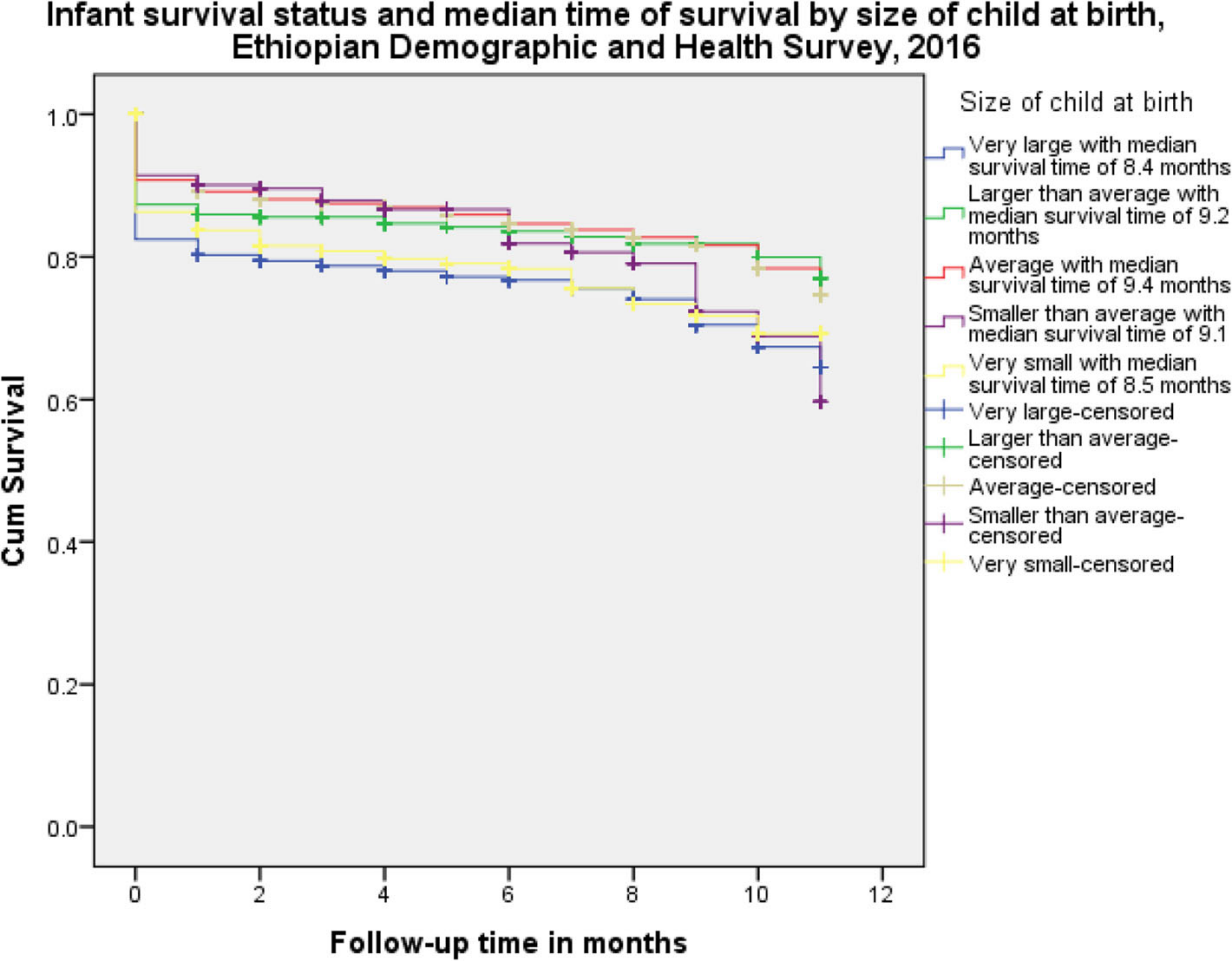
Death of infants by months in Ethiopia using 2016 Ethiopian Demographic and Health Survey

The estimated hazard ratio for multiple births in relation to singleton was 4.07(95%CI:1.14–14.5). The risk of death for multiple births was 4.07 times higher than singleton. With regard to average size infants, the estimated hazard ratios for very small size infants and smaller than average size infants were 3.74 (95% CI: 1.73–8.12) and 3.23 (95% CI: 1.40–7.41) respectively. The risk of infant death for very small size and smaller than the average size were higher by 3.74 and 3.23 times compared to the average size of infants. Similarly size at birth was significantly associated with infant mortality (Fig. 3). In relation to female infants (the reference category), the estimated hazard ratio for male infants was 1.26 (95% CI:1.01–1.56). It implied that the risk of death of infants was 1.26 times more likely than for female infants (Table 2). For the final multivariate regression cox model, the fitness statistic was checked by performing log-log survival curves based on Schoenfeld residuals and the model fitness statistics was significant (*P* = 0.0137).

## Discussion

Currently, saving the lives of infants and their mothers is a global priority [14]. According to the National Strategy (2015/16–2019/20) for Newborn and Child Survival, Ethiopia has envisioned to end all preventable newborn and child deaths by 2035. Specifically, it aims to reduce infant mortality rate from 44/1000 live births (2013 level) to 20/1000 live births. The strategy identified and prioritized 39 high impact and cost effective newborn and child survival interventions with key guiding principles for implementation of the strategy including equity and accessibility; community engagement, empowerment and ownership; integration; partnership; efficient use of resources; innovation and use of technology; evidence based decision making; and provision of quality MNCH services [15]. To support this ambitious strategy with evidence, we conducted this study on the proximate determinant of infant mortality in Ethiopia, using 2016 Ethiopian Demographic and Health Surveys (EDHS) with the application of the Cox regression hazard model. The analyses revealed that Distal/socioeconomic factors (educational level of mothers), intermediate/mother’s characteristics (preceding birth intervals) and Proximal/child’s characteristics (plurality, size of child at birth and sex of the child) were statistically significant to infant mortality in Ethiopia.

In this study, about 4.8% of infants died before their first birthday (Table 1). Infant were most at risk during the early months of life which is when approximately, 65% of infants deaths occurred (Fig. 2). We found that the infants mortality rate in Ethiopia is relatively higher than the one reported in Tanzania [16]. In Tanzania, about 96% of infants survive their first year, and 56% of infant deaths occur during the neonatal period. This difference could be due to poorer antenatal care practices, and short follow up time for the infants [14–17].

In this study, infants of mothers with a higher level of education or secondary education had a lower risk of infant mortality (32 and 41% respectively) compared to the reference category. Another study conducted in Ethiopia [18] suggested that neonates born to women with secondary or higher schooling versus no education had a lower risk of dying. A study conducted in Bangladesh [19] suggested that mother’s education was significantly associated with child survival. A study by Mustafa et al. showed that Kenyan mothers with a higher level of education have a better socioeconomic status, tend to be more aware of good childcare practices and their child’s health status [20]. In this study, the risk of infant death with a preceding birth interval shorter than 24 months and longer than 47 months was higher (Table 2). This finding is consistent with a study conducted in Nigeria in which the risk of death for infants born with birth interval less than 2 years was higher [21]. This is consistent when compared to findings from Nigeria [21], in which infants of mothers with a higher level of education were less at risk of dying during infancy compared to the reference category.

This study also demonstrated that, the risk of death of infant was higher among multiple births than singleton. A previous study conducted using the EDHS 2011 [22] suggested that multiple births were more likely to die than singletons. Another study conducted in southwest Ethiopia [23] also suggested that twins, were much more likely to die than single births, even after taking their birth weight into account. One possible explanation for this observed association could be [24] multi-fetal pregnancy and multiple births, including twins and higher order multiples such as triplets and quadruplets were at high-risk during both pregnancy and birth. These high-risk pregnancies were frequently accompanied by a number of associated fetal and neonatal complications that required special and expensive medical care.

With regard to birth interval, the risk of infant mortality was higher for infants with a preceding birth interval shorter than 24 months and lower with preceding birth interval longer than 47 months. The mean estimate of survival time for infants who were born within less than 24 months was 6.2 (95% CI, 5.8–6.5) months, whereas for infants born above 47 months of preceding birth interval, it was 5.5 (95% CI, 5.22–6.8) months. The difference in time between the two categories of infants was statistically significant (*p* < 0.001). A study in Global infant mortality trends and attributable determinants – an ecological study using data from 192 countries for the period 1990–2011 [25] infants born after longer interpregnancy intervals had better odds of survival. A study in Tanzania [16] showed that shorter birth interval increased infant and child mortality. Similarly, another study from Malawi [26] indicated that the effect of birth interval was largely limited the infant mortality. A study in Ethiopia [27] also indicated that birth interval is significantly associated with infant mortality. This could probably be attributed to biological factors: giving a second birth within such a short birth interval affects the health of both child and mother. A shorter length of the birth interval may negatively affect maternal health, increase the susceptibility of infectious diseases, and cause familial resource competition among children [28, 29].

In this study, the number of infants born with low birth weight was much higher as compared to other studies [26, 29] and this study also showed that the size of the child at birth was significantly associated with infant mortality. This variation might be due to the recall bias that might be introduced into this study because the size of the child at birth was assessed by mothers self-report. The risk of death for infants with very small size and smaller than the average size were higher. A study in Bangladesh [30] showed that small birth size was associated with a higher infant mortality rate. Poor nutritional status may influence size at birth and thereby could affect the risk of infant mortality as well [31, 32].

Our findings showed that, the sex of the infants was a significant risk factor for infant mortality in Ethiopia and male infants had the highest risk of deaths compared to female infants. Another study in Ethiopia [18] showed that male children are at greater risk of dying before reaching their first birthday. This is also supported by other studies in which infant mortality rate was higher for males than females [33–35]. The fact that girls have a biological advantage against many causes of death than boys can be a possible explanation of the higher risk of male child deaths [18, 28, 36, 37], which is due to a lesser vulnerability to perinatal conditions, congenital anomalies, and infectious diseases [38].

This study had the following limitations. Some of the variables were measured on subjective basis or self-report of the study participants, thus it can introduce bias. For example the size of child at birth was assessed by self-report; which might have introduced bias on the actual size of the child at birth. Consequently, the number of infants with low birth weight was higher in this study. Failure to consider time period effects or years of birth into regression might be another source of limitation. The changes of guidelines including the National Strategy for Newborn and Child Survival in Ethiopia between 2011 and 2015 might affect differently babies that were born in 2011 vs babies born in the later period. Inaddition, exclusion of observations with incomplete/missing data might have had an effect on the strengths of the associations that we observed, as well as on the accuracy of our estimates as indicated by their confidence intervals.

## Conclusion

A significant infant mortality rate was observed with more than two-third of infant deaths during the first months of life. It is found that multiple birth, preceding birth interval shorter than 24 months, preceding birth interval longer than 47 months, very small size of the infant at birth, smaller than average size of the infant at birth and being a male sex of the infant was significantly associated with infant mortality.

The need to strengthen and monitor for the comprehensiveness of maternal and child health care services (including ANC folloup, PNC follow-up, family planning and immunization) during the earlier periods of infancy is highly recommended. Moreover, mothers with multiple birth, giving birth to a child with birth interval of below 2 years and infants born with smaller than average size at birth deserve special attention.

## Data Availability

The data sets used and/or analyzed during the current study are available in the Ethiopian statistical agency and ministry of health.

## Abbreviations

ANC: Antenatal Care
CI: Confidence Interval
EDHS: Ethiopian Demographic and Health Survey
MEASURE DHS: Monitoring and Evaluation to Assess and Use Results Demographic and Health Surveys
PNC: Postnatal Care
RR: Relative Risk
SNNPR: Southern Nations, Nationalities, and Peoples’ Region
